# Extracellular Vesicles from Mesenchymal Stem Cells: Potential as Therapeutics in Metabolic Dysfunction-Associated Steatotic Liver Disease (MASLD)

**DOI:** 10.3390/biomedicines12122848

**Published:** 2024-12-14

**Authors:** Xue Zou, David Brigstock

**Affiliations:** 1 Center for Clinical and Translational Research, The Abigail Wexner Research Institute at Nationwide Children’s Hospital, Columbus, OH 43205, USA; xue.zou@nationwidechildrens.org; 2 Department of Surgery, Wexner Medical Center, The Ohio State University, Columbus, OH 43212, USA

**Keywords:** metabolic dysfunction-associated steatotic liver disease, metabolic dysfunction-associated steatohepatitis, extracellular vesicles, exosome, mesenchymal stem cell, fibrosis

## Abstract

**Background/Objectives:** Metabolic dysfunction-associated steatotic liver disease (MASLD) is characterized by the accumulation of triglycerides within hepatocytes, which can progress to more severe conditions, such as metabolic dysfunction-associated steatohepatitis (MASH), which may include progressive fibrosis, leading to cirrhosis, cancer, and death. This goal of this review is to highlight recent research showing the potential of mesenchymal stem cell-derived extracellular vesicles (MSC-EVs) in reducing the key pathogenic pathways of MASLD or MASH. **Methods:** Relevant published studies were identified using PubMed with one or more of the following search terms: MASLD, MASH, NAFLD, NASH, exosome, extracellular vesicle (EV), therapy, and/or mesenchymal stem cells (MSC). The primary literature were subsequently downloaded and summarized. **Results:** Using in vitro or in vivo models, MSC-EVs have been found to counteract oxidative stress, a significant contributor to liver injury in MASH, and to suppress disease progression, including steatosis, inflammation, and, in a few instances, fibrosis. Some of these outcomes have been attributed to specific EV cargo components including microRNAs and proteins. Thus, MSC-EVs enriched with these types of molecules may have improved the therapeutic efficacy for MASLD/MASH and represent a novel approach to potentially halt or reverse the disease process. **Conclusions:** MSC-EVs are attractive therapeutic agents for treating MASLD/MASH. Further studies are necessary to validate the clinical applicability and efficacy of MSC-EVs in human MASH patients, focusing on optimizing delivery strategies and identifying the pathogenic pathways that are targeted by specific EV components.

## 1. Introduction

Metabolic dysfunction-associated steatotic liver disease (MASLD), previously termed non-alcoholic fatty liver disease (NAFLD), affects about 25% of the global population and is characterized by ≥5% liver steatosis without any concurrent liver disease, plus the presence of at least one of five cardiometabolic risk factors [1]. MASLD may progress to a more severe condition, termed metabolic associated steatohepatitis (MASH; previously termed non-alcoholic steatohepatitis (NASH)), that is characterized by liver inflammation and fibrosis, the latter of which is a predictor of disease mortality and which can progress severely, resulting in cirrhosis, cancer, loss of critical hepatic function, and the need for liver transplantation [2]. While exercise and diet modifications are strongly implicated in improving outcomes, there is a significant level of non-compliance, and alternative treatment strategies are required [3].

The purpose of this review is to summarize the pre-clinical evidence showing that extracellular vesicles (EVs) from mesenchymal stem cells (MSC) are emerging as promising agents with which to treat MASLD/MASH. EVs are nano-sized bilayer lipid membrane particles that contain a complex molecular cargo comprising RNA, protein, lipids, etc. [4,5]. EVs have become recognized for their pivotal roles in intercellular communication because they are released extracellularly and are transported in body fluids (interstitial fluids, blood, lymph) whereupon they may bind and be taken up by other cells into which their molecular contents are released, effecting a transfer of molecular information from EV producer cells to recipient cells [6,7,8,9,10,11,12,13,14]. As a result, recipient cells may undergo transcriptional, functional and/or and phenotypic changes according to the bioactive components received. This natural delivery mechanism has been exploited as a means of drug delivery using EVs that are modified to carry specific therapeutic agents or that contain cargo components that are naturally therapeutic [15,16,17]. Such is the case with MSC-EVs which have been shown to have intrinsic therapeutic, reparative, or restorative actions in numerous disease models [18] and which are being widely tested in human clinical trials [19]. Moreover, EVs have been shown to be non-immunogenic and to have low toxicity in many in vivo studies [20,21,22], as well as having strong tissue-homing and cell uptake capabilities, and avoiding the concerns associated with cell (i.e., MSC)-based therapy, such as tumor formation and low seeding efficiency. In this article, we review promising data showing that the steatosis and inflammatory components of the MASLD/MASH are effectively reduced by MSC EVs and that, in some cases, fibrosis is reduced as well. The relevant literature were identified using PubMed with one or more of the following search terms: MASLD, MASH, NAFLD, NASH, exosome, EV, therapy, and/or MSC.

## 2. Metabolic Dysfunction-Associated Steatotic Liver Disease (MASLD)

Liver diseases cause over 2 million deaths every year, accounting for 4% of the total number of deaths worldwide [23,24]. About 25–30% of the global population is affected by metabolic dysfunction-associated steatotic liver disease (MASLD), which is increasing in prevalence and which has serious disease sequelae [25,26,27,28]. The criteria for the diagnosis of MASLD are ≥5% hepatic steatosis without any concurrent liver disease, plus the presence of at least one of five cardiometabolic risk factors [1]. The term MASLD was introduced in 2023 to replace “non-alcoholic fatty liver disease” (NAFLD), which had the same standard (≥5%) for steatosis and which required an absence of significant alcohol usage for diagnosis, but did not take cardiometabolic risk factors into account [1]. Metabolic dysfunction associated steatohepatitis (MASH), previously called non-alcoholic steatohepatitis (NASH), is a severe form of MASLD that involves liver inflammation and fibrosis which may lead to severe debilitating and life-threatening conditions, including cirrhosis and liver cancer. With MASH estimated to be present in ~25% of all MASLD patients [29], it is on track to become the leading cause of liver transplantation and is a rising cause of liver cancer [29,30,31]. The key features of MASH are steatosis, hepatocyte ballooning, and inflammation. The inflammatory process occurs in response to chronic insults to the liver (e.g., metabolic disturbances, insulin resistance, obesity, diabetes) which cause lipotoxic- or ER-stress in hepatocytes [32,33], the production of damage-associated molecular patterns from dying hepatocytes, the production of inflammatory cytokines from hepatocytes or Kupffer cells, and the infiltration of monocyte-derived macrophages and neutrophils [34,35,36,37,38]. These profound cellular and environmental changes may trigger the activation of HSC, resulting in their production of insoluble collagen and other ECM molecules which are unrelentingly deposited in the interstitial spaces, leading to progressive fibrosis and gradual inexorable hepatic decline. In its advanced stages, MASH livers are highly cirrhotic and liver transplant is usually the only option for survival [39,40,41].

Liver fibrosis in MASH has become a major research focus because its severity is the best predictor of disease mortality [42,43,44]. Several clinical studies have shown that reduced body weight resulting from modified diets or bariatric surgery is associated with improved MASH histological and fibrosis scores [45,46,47,48]. Thus, while a worthwhile treatment for MASLD/MASH patients is for them to lose weight by modifying their diet and increase their exercise, most individuals are non-compliant even though a weight loss of ~10% is sufficient to reduce steatohepatitis and is effective in improving liver fibrosis [45,49]. In light of this difficulty, there is intense interest in developing therapeutic strategies that reduce or prevent the production or accumulation of fibrotic scars, for which a very large variety of cellular and molecular targets are becoming recognized [50]. There is new hope with the recent approval by the FDA of the thyroid hormone agonist resmetirom (Rezdiffra^TM^), which reduces fibrosis in MASH and reduces MASH without increasing fibrosis, but optimism is tempered by an improvement in only 50% of patients in Phase III trials [51]. While other drug candidates are still under evaluation, there remains the possibility that EVs can be harnessed for their therapeutic potential and bring new possibilities for MASLD/MASH treatment.

## 3. MSC Extracellular Vesicles (MSC-EVs)

### 3.1. Properties of MSC

Mesenchymal stem cells (MSC) are a type of undifferentiated adult stem cells, which in adults, can self-renew after tissue injury and be programmed, in response to environmental signaling cues, to differentiate along specific developmental pathways into a variety of cell types in various tissues and organs [52,53]. MSCs can be derived from various sources, including from teeth [54], peripheral blood [55], umbilical cord tissue [56], bone marrow [57,58], embryonic tissue [56,59], placenta [56], periosteum [60], umbilical cord tissue [61], and adipose tissue [62]. MSCs can be differentiated into different tissues, support hematopoietic stem cells [63], and regulate the immune system [64]. In light of their regenerative potential, MSCs have great potential for the therapy of neurological diseases [65], cardiovascular diseases [66], cancer [67], autoimmune diseases [68] and virus infections (e.g., COVID-19) [69]. In clinical studies of rheumatoid arthritis, the transplantation of MSCs into the diseased joint reduced inflammation by interacting with immune cells and their secreted factors [70,71]. Cancer studies have shown therapeutic effects of MSCs in hematopoietic and lymphoid cell neoplasms, acute leukemia, and pancreatic cancer, among others [72,73]. However, the transplantation of MSCs carries certain risks. For example, the intra-arterial administration of MSCs led to myocardial micro-infarction [74], while MSCs may undergo transformation and result in the production of undesirable cell types, including those that cause cancer [75,76,77,78]. Additionally, there are numerous challenges relating to the mass-scale production of high-quality MSCs for in vivo therapeutic applications. Further, the heterogeneity of MSCs resulting from donor variations (e.g., age, inflammatory disease state) can have a high impact on therapeutic efficacy. Depending on their tissue of origin, 3–5 × 10^6^ cells/kg of donor-derived CD34+ cells are required to achieve therapeutic efficacy [79]. These drawbacks have severely hampered the clinical applicability of MSC-based therapy, and have favored studies of cell-free MSC-based treatments.

### 3.2. General Features of EVs

**Figure 1:**
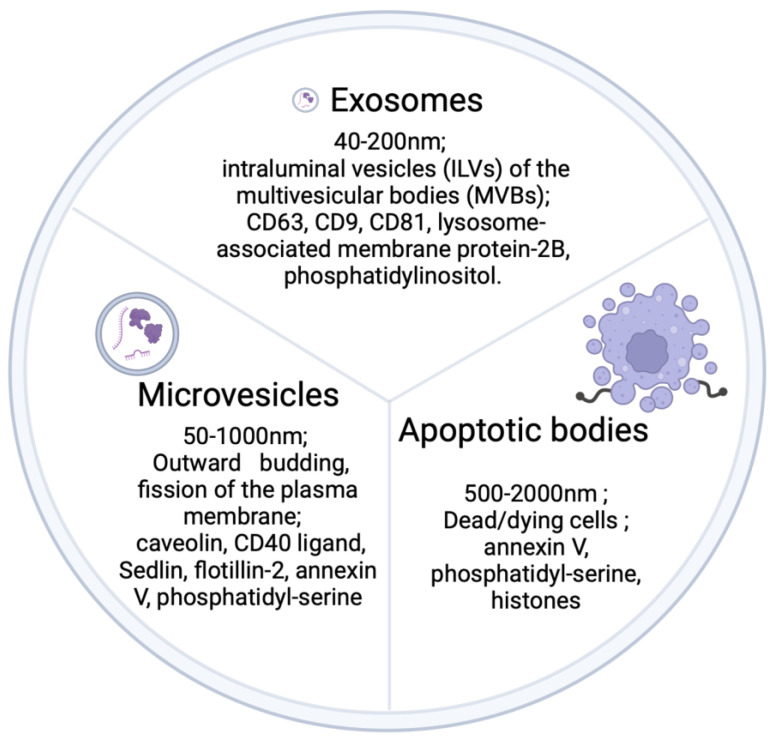
Schematic depiction of the principal EV subtypes.

**Figure 2:**
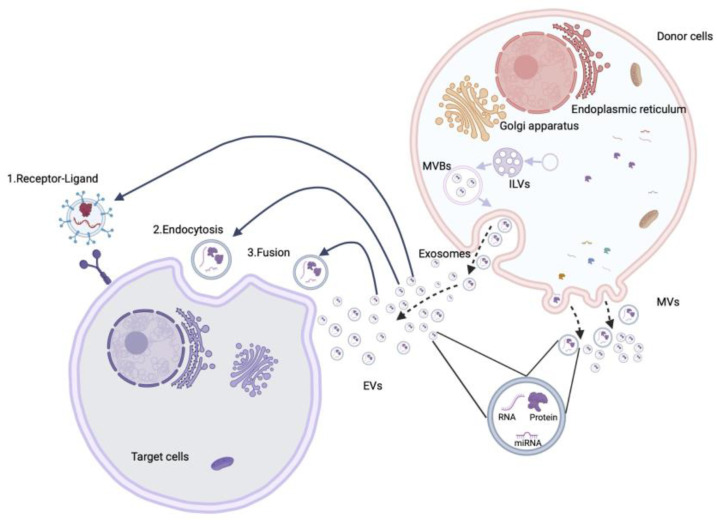
EV biogenesis, release, and uptake. Internal budding of early endosomes results in the formation of intraluminal vesicles (ILVs) that contain cytosolic components (proteins, mRNAs, miRs). ILVs are contained within multivesicular bodies (MVBs) that may fuse with the plasma membrane, resulting in the liberation of their vesicles into the extracellular space, at which point the vesicles become exosomes. Microvesicles (MVs) are formed by the budding of the plasma membrane, resulting in the vesicular entrapment of free cytoplasmic components and the liberation of the MVs into the extracellular space. Both populations of vesicles (exosomes, MVs) are collectively termed EVs and are challenging to discriminate from each other once they have been released from the producer cells. The molecular cargo in EVs may be delivered to target cells by a variety of mechanisms that may involve fusion, endocytosis and/or receptor–ligand interactions.

First described more than 50 years ago [80], EVs are nano-sized membrane-limited particles that contain a broad variety of cell-derived materials, including proteins, lipids, RNA and DNA [81]. Three categories of EVs have been generally recognized: (i) apoptotic bodies, approx. 500–2000 nm in diameter, which are produced by dead or dying cells and characteristically contain annexin V, phosphatidyl-serine, and histones; (ii) microvesicles (MVs), approx. 50–1000 nm in diameter, which are characterized by markers such as caveolin, CD40 ligand, Selin, flotillin-2, annexin V, and phosphatidyl-serine; and (iii) exosomes, approx. 40–200 nm in diameter, which contain membrane proteins such as CD63, CD9, CD81 and lysosome-associated membrane protein-2B or glycosyl phosphatidylinositol (Figure 1). A major difference between microvesicles and exosomes is their manner of biogenesis [82]. MVs originate by budding off from the plasma membrane into the extracellular space while exosomes are initially formed as intraluminal vesicles (ILV) by the internal budding of early endosomes, resulting in the production of multivesicular bodies (MVB) (Figure 2). Whereas some MVBs are degraded by fusion with lysosomes, other MVBs fuse with the plasma membrane, resulting in the release of their internal vesicles into the extracellular space, at which point they become exosomes.

Although exosomes and microvesicles were initially considered to be a means for removing cell waste products [80], the work over the last 15–20 years has firmly established that they are involved in cell–cell communication by virtue of their ability to shuttle their complex molecular payloads between different cells (Figure 2). EVs can deliver their molecular cargo into target cells by multiple mechanisms [83] that include the following: (a) the fusion of the EV membrane with the plasma membrane of target cells, resulting in the direct release of EV cargo into the cytoplasm; this is the most direct mechanism of EV uptake and is affected by alterations in the extracellular environment (pH, temperature) as well as membrane lipid composition [84]; (b) clathrin-mediated endocytosis, a process that involves over 60 different proteins and which is characterized by the binding of EVs to the clathrin-enriched plasma membrane and subsequent entry into the cell in clathrin-coated transport vesicles, a process that is dependent on dynamin 2; (c) micropinocytosis, a process by which EVs interact with invaginated membrane ruffles that then internally pinch off, resulting in the internalization of EVs; this is dependent on the actin cytoskeleton, phosphatidylinositol 3-kinase activity, and dynamin-2 [85]; and (d) the interaction of EVs with lipid rafts which are organized plasma membrane microdomains [86] that are enriched in cholesterol, glycosphingolipids, and glycosyl-phosphatidylinositol (GPI)-anchored proteins [83]. Such EV uptake is reduced in cells treated with the cholesterol-reducing agents MbCD [87], filipin [88] or simvastatin [87].

The shuttling of EVs between producer and recipient cells represents an important mechanism by which complex signaling information can be transferred among different cells. While this likely contributes to the regulation of homeostasis, much attention has been focused on the significance of EVs in driving pathogenic sequalae and disease progression. For example, EVs have important roles in virus infection because viral components (RNA, DNA, protein) may be packaged into EVs and then shuttled into non-infected cells [89,90,91,92]. Also, EVs (exosomes) share the same pathway for released enveloped retrovirus particles (e.g., HIV) and can carry viral protein and viral RNA, such as Nef [93] and Gag [94]. In some cases, this type of communication can result in the production of viral particles [95]. In cancer, EV-mediated cell–cell communication regulates the tumor microenvironment. For example, EVs from multiple myeloma cells possess NKG2D ligands that are able to activate NK cells and stimulate their functions [96]. Cancer EVs can help establish tumor cell settlement and metastasis [97].

In view of their role as natural molecular-delivery vehicles, much attention has been focused on the exploitation of this function for the development of new drug delivery systems. There are several features of EVs that favor this approach. First, EVs are relatively immunologically inert and non-toxic, which avoids common problems with other drug delivery vehicles that can cause super sensitivity, cytokine release syndrome, biological deactivation, and other immune reactions [98], Second, EVs have inherent targeting and homing ability and can cross the blood-brain barrier. Third, EVs provide a stable environment that protects their cargo from extracellular degradation. Fourth, EVs are amenable to modifications, such as the incorporation of improved or new therapeutic payloads or alterations in their membrane biochemical composition to improve their targeting to existing cells or to allow for entirely new targeting opportunities. For example, this may involve changes in the composition or relative proportions of EV surface proteins that interact with target cells (e.g., integrins, HPSG, LRP, LFA-1, ICAM-1, CD81, CD9 [99,100]) or the incorporation of novel membrane components.

### 3.3. MSC-EVs

MSC-EVs carry markers of EVs (e.g., CD81, CD63, Tsg101 [101]) and of MSCs (e.g., CD29, CD73, CD90, CD44 [102]). MSC-EVs possess intrinsic therapeutic properties which are reflective of the regenerative and therapeutic actions of the MSCs from which they are derived [103,104,105,106]. Evidence suggests that MSC-EVs may be more potent than MSCs themselves. MSC-EVs play an important role in the regulation of the immune system [107] and they modulate the balance between the immune response and immune tolerance [108,109]. MSC-EVs significantly reduce the immunomodulatory potency of T cell receptor (TCR)- or Toll-like receptor 4 (TLR4)-stimulated splenocytes, and TGF-β1, PTX3, let-7b-5p, and miR-21-5p in MSC-EV cargo contribute to the suppressive effect of the EVs in Th1 and Th17 cytokine production [110]. In a recent cancer study, anti-CD3/CD20 bispecific antibodies carried by MSC-EVs activated cytotoxic T lymphocytes and the release of TNF-α and interferon-γ in a CHS model of B cell lymphoma. On the other hand, MSC-EVs contain miR-15a/miR-16, which down-regulated bcl-2 expression and caused apoptosis [111].

These examples highlight the potential application of MSC-EV therapy, which is buttressed by studies of their safety and efficacy, especially as compared to MSC. The significant risk of causing tumors through the administration of MSCs is essentially negated using MSC-EVs [112]. Also, when MSCs were administered intravenously, they became trapped in the pulmonary capillary network, causing pulmonary embolism [77]. In contrast, EVs are able to pass through blood-brain barriers and the placenta easily [21,113,114], and there are many instances of successful MSC-EVs therapy after intravenous administration [115]. The therapeutic use of MSC-EVs in murine models of asthma resulted in reduced inflammation, goblet cell hyperplasia, and airway hyper-responsiveness, and these outcomes were associated with the EV-mediated suppression of the Wnt/β-catenin pathway [116]. Bone marrow MSC (BM MSC) EVs improved fibrocartilage regeneration at the tendon–bone interface and decreased supraspinatus fatty infiltration in a murine rotator cuff repair model by promoting chondrogenesis and anti-adipogenesis, which was primarily attributed to the delivery of EV miR-140 [117]. In a rat intra-uterine adhesion model, adipose-derived MSC-EVs improved pregnancy outcomes by enhancing endometrial thickness and gland frequency, decreasing the extent of fibrosis, decreasing conception time, and increasing implantation frequency; these improvements were associated with the enhanced expression of integrin-β3, LIF, and VEGF [118]. The engraftment of MSCs is documented to be quite inefficient and had been considered to be insufficient in accounting for their therapeutic effect, which has been attributed to their paracrine action [119], which likely involves their local release of EVs.

Whereas the viability of MSCs may be compromised by storage [120], purified EVs can be stored for very prolonged periods (e.g., >8 months at −80 °C ) without the loss of structural integrity or biological activity [121]. Additionally, the acquisition of hundreds of millions of cells for MSC treatment typically takes around 10 weeks in cell cultures, which is both time-consuming and costly compared to EV collection [122]. EVs can be obtained continuously and efficiently in large quantities [73]. Thus, as compared to MSC, the use of MSC-EVs for therapy is safer, more effective, and more economical [123].

## 4. MSC-EV Therapy in MASH-like Models

### 4.1. Human Umbilical Cord MSC (hUC MSC) EVs

Human umbilical cord-derived MSCs (hUC MSCs) are usually obtained from Wharton’s jelly or the interior aspect of the umbilical cord. hUC MSCs have certain advantages over other MSC sources, such as involving fewer ethical concerns, pain-free collection from abandoned umbilical cords, and the absence of immune rejection issues [124]. Additionally, their ability to modulate immune responses makes hUC MSCs a promising option for allogeneic transplantation without the need for immunosuppression [125]. hUC MSC EVs are produced at a high frequency and have broad therapeutic properties akin to those of their producer cells, such as the promotion of cell migration and survival, wound repair, neuroprotection, and regulation of inflammation [126,127,128].

When hUC MSC EVs were administered weekly (i.v.; 20 mg/kg) to C57BL/6J mice on a 6-week methionine-choline-deficient (MCD) diet, the mice exhibited less body weight reduction, improved AST and ALT, and reduced steatosis and hepatocyte ballooning as compared to control mice on an MCD diet but not receiving EVs [129]. These improvements were accompanied by an EV-mediated reduction in MCD-induced plasma TNFα, IL-6 or IL-1β levels and partial reversal in the liver of their presumptive transcriptional regulator, phosphorylated NF-κB (p65). As compared to control mice, hUC MSC EV administration in MCD-fed mice was also associated with an increased frequency of anti-inflammatory macrophages (CD206^+^, Arg-1^+^, Il-10^+^) and the restored expression of peroxisome proliferator-activated receptor alpha (PPARα), a master regulator of lipid homeostasis [129]. In vitro, hUC MSC EVs reduced foaming and the production of TNF-α, IL-6 and IL-1β by RAW264.7 macrophages that had been treated with oxidized low density lipoprotein (ox-LDL), as well as reduced PPARα expression in HepRG or Huh1-6 hepatocytes [129].

In another study, C57BL/6J mice received either a 20-week high fat high cholesterol (HFHC) diet for 20 weeks with hUC MSC EVs (100 µg/mouse, i.v.) administered every three days for the last six weeks, or an MCD diet for 4 weeks with hUC MSC EVs (100 µg/mouse, i.v.) administered twice a week for the last two weeks [130]. As compared to non-EV-treated animals, the hUC MSC EV treatment of animals on either of the injurious diets consistently resulted in restored serum AST and ALT levels, reduced steatosis, the reversal of lipid-related gene expression (SREBP-1c, PPAR-α, Fabp5, CPT1α, ACOX, FAS), and a reduced frequency of F4/80 macrophages in the liver, while also causing the macrophages to de-polarize, favoring a reduced CD11^+^ M1 pro-inflammatory phenotype and an increased CD206^+^ M2 anti-inflammatory phenotype [130]. Additionally, consistent with also exerting antioxidant actions, hUC MSC further inhibited MCD- or HFHC-induced levels of MDA, Cyp2E1 and ROS, increased the activity of antioxidant SOD and GSH, and increased the expression of the Nrf2/NQ0-1 antioxidant transcription pathway, the latter of which was confirmed to be involved by in vitro studies in which the therapeutic increase in Nrf2 or NQ01 was brought about by hUC MSC EVs in palmitic acid- or MCD-treated hepatocytes blocked by the NRf2-specific blocker, ML385 [130].

Similar conclusions were drawn when hUC MSC EVs were added to primary mouse hepatocytes in vitro during the treatment of the cells with palmitic acid (PA), with the result that hUC MSC EVs reduced PA-stimulated lipid deposition, ROS production, as well as the production of numerous molecules involved in FA synthesis, oxidative stress, and inflammation, as shown by rescued transcripts levels for ACCα, FASN, SCD1, PPARγ, NOX 2, NOX 4, TNF-α, IL-6, IL-β, and CCL2 or by the rescue of ASN, PPARγ, p-IκBα, p–NF–κB, Keap-1, NOX2, Nrf2, and HO proteins [131]. When C57BL/6 mice on a 16-week high fat diet (HFD) were administered with hUC MSC EVs weekly (120 µg/mouse, i.v.), the mice showed corrected body weights, liver weights, blood glucose and insulin, and serum AST or ALT, reduced HFD-mediated steatosis, hepatocyte ballooning, macrophage inflammation, a reduced production or expression of ROS, TNFα, IL-6, ACCα, FASN, SCD1, PPARγ, NOX2, NOX4, TNF-α, IL-6, IL-1β and CCL2, and restored levels of antioxidant SOD and CAT. Numerous therapeutic outcomes in vitro were lost when EVs were used from hUC MSC that had been transfected with a mi-24-3p inhibitor, resulting in the finding that EV therapy was mechanistically linked to the delivery of EV miR-24-3p, which suppressed lipid accumulation, ROS production and inflammatory responses in PA-treated hepatocytes through its targeting of Keap-1 [131].

hUC MSC EVs were similarly therapeutic when administered (10 µg/g per week) to C57/Bl6 mice over the last 4 weeks of a 14-week HFD feeding [132]. EV-treated mice showed reduced levels of steatosis, liver damage (AST, ALT), liver triglycerides, total cholesterol, hepatic expression of TNF-α, IL-1, and IL-6, and restored insulin sensitivity. In vitro, the addition of the EVs to oleic-palmitic acid (OPA)-treated L02 human hepatocytes decreased lipid accumulation in the cells, which was attributed to the increased FA β oxidation and inhibition of fatty acid synthesis. Differential mRNA analysis showed that, both in vivo or in vitro, there was a consistent EV-mediated upregulation of the PPARα/CPT-1A pathway to promote fatty acid oxidation and the suppression of the SREBP-1c/Fasn pathway to reduce fatty acid synthesis, and these outcomes were mechanistically shown to be mediated by EV-delivery of CAMKK1, an activator of AMP-activated protein kinase (AMPK), an upstream regulator of PPARα and SREBP-1c [132].

hUC MSC EVs have also been reported to be enriched in mir-627-5p, which was causally linked to the ability of the EVs, when added to PA-stimulated human liver L-02 cells, to promote cell viability and the expression of G6Pc, PEPCK, and SREBP-1c while reducing apoptosis and the expression of PPARα and FTO (fat mass and obesity-associated gene), the latter being a direct target of EV-derived miR-627-5p. The inhibition of FTO by miR-627-5p was linked to the EV-stimulated improvement in cellular glucose and lipid metabolism [133]. In a high fat high fructose NAFLD model in rats, the administration of hUC MSC EVs reduced diet-induced increases in body and liver weights, steatosis, insulin tolerance and liver injury, all of which were attributable to the targeting of FTO by miR-627-5p [133].

Overall, more therapeutic investigations have been undertaken with hUC MSC EVs in NAFLD models than with any other source of EVs. The data described above show a remarkable consistency in outcomes despite the use of different animal models and various modes of EV administration and dosing. While multiple downstream pathways and targets have been studied, an emerging theme is that hUC MSC EVs have a broad and consistent ability to regulate steatosis, lipid metabolism, oxidative stress, and inflammation. A drawback in these studies is that readouts of fibrogenesis and fibrosis have not been studied, likely because some of the models do not result in a robust fibrotic response.

### 4.2. Bone Marrow MSC (BM MSC) EVs

Bone marrow is contained within bone cavities and contains hematopoietic stem cells and MSC. The therapeutic potential of adult BM MSCs for bone repair has been widely studied because the cells can differentiate into osteoblasts, chondrocytes, or adipocytes [134]. BM MSCs may contribute to bone regeneration through various mechanisms, including the direct differentiation into bone cells, recruitment of other regenerative cells, or by creating a supportive environment through the secretion of trophic growth factors [135]. The secretome of BM MSCs includes a large variety of cytokines and growth factors (e.g. VEGF, TGF-β, IGF-1, HGF, angiopoietins), as well as EVs, which collectively contribute to bone regeneration, survival, and angiogenesis [136,137,138,139].

In a 12-week HFD regimen in rats, the administration of BM MSC EVs over the last 6 weeks (15–120 µg/kg twice a week) resulted in suppressed steatosis, hepatocyte ballooning, serum AST and ALT, and the expression of SREBP-1, SREBP-2, ACC (fatty acid synthesis) or CD36 (lipid uptake), decreased NAFLD activity score (NAS), and an increased expression of PPARα and CPT1 (fatty acid oxidation) [140]. The EVs were shown to induce a 10-fold increase in hepatic miR-96-5p expression and a decrease in the expression of its caspase-2 target, although functional evidence for a mechanistic link between these molecules was not presented. However, consistent with the suppression of caspase 2, HFD-induced hepatocyte apoptosis assessed by Bax and Bcl2 expression was normalized in EV-treated livers. Finally, the suppression of mitophagy (autophagic degradation of damaged mitochondria) in the NASH model, as shown by the reduced expression of PINK 1, Parkin, ULK1, BNIP3L, ATG 5, ATG 7 and ATG 12, was reversed by BM MSC EV administration, showing that EVs restored mitochondrial regulatory mechanisms that are perturbed during NASH [140].

### 4.3. Adipose MSC (AD MSC) EVs

Adipose-derived mesenchymal stem cells (AD-MSCs) are easily isolated from body fat under the skin, representing one of the richest sources of stem cells in the body. AD-MSCs exhibit pluripotent lineage characteristics and can be cultured for long periods of time without losing their stem cell properties. Additionally, AD-MSCs are HLA-DR negative, allowing for allogeneic transplantation without the need for immunosuppression [141]. AD-MSCs can be differentiated into adipocytes and have superior adipogenic properties but can also be differentiated into chondrocytes or osteoblasts to drive, respectively, chondrogenesis or osteogenesis. The AD-MSC secretome contains numerous regeneration-promoting components, including EVs which themselves can regulate adipocyte function and obesity sequelae [142]. Human AD MSC EVs contain mitogen kinases and cell regulatory components such as PI3K-AKT, JAK-STAT, and Wnt, which have been implicated in the improvement in urethral function in animal models of stress urinary incontinence [143].

EVs from AD MSCs were shown to contain naturally high levels of miR-223-3p and caused the intracellular concentration of miR-223-3p to become elevated in NCTC1469 normal mouse hepatocytes [144]. When AD MSCs were transduced with a lentiviral miR-233-3p mimic, the EVs produced by the cells were 4-fold enriched in miR-233-3p and, when added to NCTC cells, were more effective than control EVs in reducing pyrrolizidine alkaloids-induced lipid accumulation, triglyceride production, or total cholesterol levels, as well as the expression of TGF-β1, αSMA, and Col1a1, although the production of the latter two molecules by activated HSC, rather than hepatocytes, is more important for driving fibrosing hepatic injury [144]. When C57BL/6 mice on an 8-week HFD to induce NASH were administered AD MSC EVs i.p twice a week starting on week 2, miR-223-3p-enriched EVs were more effective than control EVs in reducing HFD-induced serum ALT and AST, as well as hepatic lipid accumulation, hydroxyproline, and fibrosis. In either experimental system, hepatocyte/hepatic levels of endogenous miR-223 were reduced by lipid-mediated injury and these were restored by EV treatment [144]. Mechanistically, the investigators showed that many of these beneficial effects were due to targeting by miR-223-3p of the E2F1 transcription factor, which has previously been implicated as a regulator of cholestatic liver fibrosis or NAFLD [145,146].

LPS-treated melanocortin-4 receptor-deficient (Mc4r-KO) mice (MC4R regulates food intake and body weight) maintained on a Western diet results in a NASH disease model that progresses rapidly and results in fibrosis within 16 weeks [147], representing a major advantage over most other rodent NASH models used in these types of studies, in which steatosis and inflammation are robust but there is often little or no fibrosis. Studies of this model were an important advancement because the treatment of the mice with AD MSC EVs over the last four weeks resulted in decreases in serum ALT, crown-like structures, and fibrosis (assessed by Sirius Red staining), as well as increases in the number of anti-inflammatory macrophages (CD11b + F4/80 + Ly6c^low^) and the expression of matrix degrading (anti-fibrotic) Mmp12 or Mmp13 [147].

These investigations are important because they showed that, in addition to EV-mediated reductions in liver injury, steatosis, and inflammation, AD-MSC EVs are also able to reduce collagen deposition into the interstitial spaces through the attenuation of expression of fibrosis-related mRNAs (TGF-β1, αSMA, Col1a1), as well as the increased production of matrix-degrading Mmps.

### 4.4. Embryonic Stem Cell-Derived MSC (ESC MSC) EVs

Embryonic stem cells (ESCs) are derived from the inner cell mass of the developing blastocyst and can be differentiated into ESC MSCs using a protocol that involves their initial development into embryoid bodies, followed by induction with high glucose and serum, resulting in cellular outgrowths comprising ESC MSCs [148]. In some studies, ESC MSCs have exhibited an improved stimulation of cell proliferation, anti-inflammatory properties and therapeutic outcomes, as compared to other types of MSCs [149,150]. This was recently shown to also be the case for their resultant EVs which, as compared to EVs from BM MSCs, cardiomyocytes, or cardiac fibroblasts, promoted superior cardiac remodeling through their ability to reduce fibrosis and increase angiogenesis in a model of cardiac ischemia reperfusion injury [151].

After streptozotocin-primed C57Bl/6J mice were administered EVs from immortalized E1−MYC 16.3 human ESC MSCs (1 or 10 µg/mouse q.o.d, i.p) over the last 3 weeks of 5-week HFD feeding, the mice, as compared to their non-EV-treated control counterparts, exhibited lower levels of steatosis, hepatocyte ballooning and inflammation and, at the 50 µg dose, significantly decreased hepatic fibrosis, as assessed by Sirus Red staining [34]. Although these EVs actually promoted the in vitro polarization of M0 to M2 macrophages and increased the frequency of CD163+ M2 macrophages in the livers of EV-treated HFD-fed mice, this pro-inflammatory pro-fibrotic cell population was nonetheless insufficient in worsening fibrosis in the face of presumably more robust pathways leading to the therapeutic outcomes addressed above and also as reflected in the reduced levels of plasma IL-6 [34].

### 4.5. Modification of MSC-EVs

As well as when using MSC EVs in their native state for therapy, treatment outcomes may be improved by modifying the EV surface chemistry or EV molecular cargo constituents to drive preferential liver homing, selective cell targeting or the spectrum of pathogenic molecular targets [152]. In one such example, EVs were isolated from hUC MSCs that had been treated with 5 µM curcumin and administered to mice (15 µg/kg, i.v.) after they had been fed a 10-week MCD diet [153]. As compared to control MSC EVs, MSC EV-cur were more effective in short- and long-term reductions in serum AST/ALT, liver triglycerides, cholesterol, hydroxyproline, oxidative stress, and the expression or production of genes related to fibrosis (αSMA, collagen-1, MMP-1), inflammation (TNF-α, IL-6 and IL-7), and apoptosis (ASK, JNK, BAX), as well as increases in lipid peroxidation and SOD activity [153]. A similar improved efficacy by MSC EV-cur versus MSC EV was seen in the reversal of PA-induced hepatic lipotoxicity, lipogenesis, expression of CD36 and PPARα, and expression of ASK1/JNK/BAX [153]. In another approach, EVs from the pan-PPAR agonist-primer induced mesenchymal stem cells (pan-PPAR-iMSC-EVs) showed a slate of improved outcomes relating to liver damage, inflammation, oxidative stress and regenerative cell proliferation and the expression of hepatocyte progenitor genes in MCD-fed mice, as well as the stimulation of PI3K/AKT in lipid-laden hepatocytes in vitro, although a comparison with EVs from non-PPAR-conditioned iMSC was lacking [154].

There are also additional supporting strategies from other non-MASH studies. For example, circDID01 is naturally present in LX2 HSC but when overexpressed in the cells by transfection, they showed reduced proliferation and activation via the stimulation of PTEN and suppression of AKT [155]. In addition, miR-141-3p, which is predicted to target circDD01, was suppressed in the ciicDID01-overexpressing cells, and when miR-141-3p was knocked down in the cells, they similarly underwent PTEN/AKT-dependent de-activation. Subsequently, the authors showed that EVs from circDID01-transfected MSC caused the suppression of LX-2 cell proliferation, decreased expression of αSMA and collagen 1, and promotion of apoptosis through the ability of EV-delivered circDID01 to bind to miR-141-3p, thereby preventing its normal pro-activation actions in the cells [155]. Although this work was restricted to an in vitro approach, it highlights the possibility of using modified MSC EVs for targeting specific HSC pro-fibrotic pathways in diseases such as MASH.

**Figure 3:**
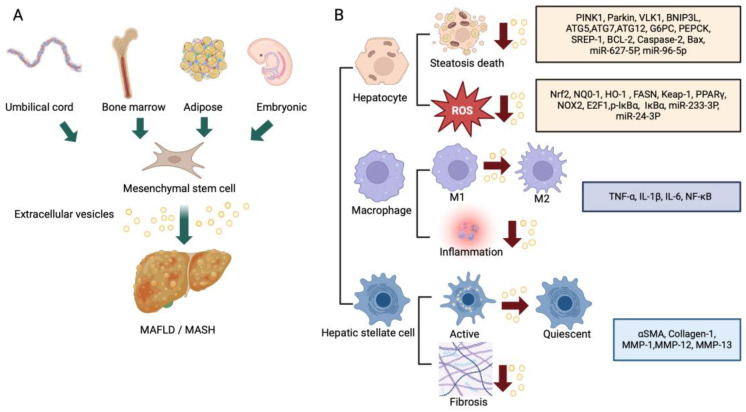
(**A**) Tissue sources of therapeutic MSC-EVs and (**B**) actions on different hepatic cell types of MSC-EVs in livers with MASLD/MASH.

The findings and features discussed above are summarized in Figure 3 and Table 1.

## 5. Unanswered Questions

The comprehensive summary above highlights the fact that multiple investigations over the past decade have shown consistent therapeutic outcomes in that most types of MSC EVs reduce MASH/NASH-like cell damage, steatosis and inflammation, as assessed using in vitro or in vivo models. However, in vitro hepatocyte models, while helpful for studying the effects of EV on lipid metabolism and the production of inflammatory mediators, is over-simplistic and does not take into account many cell–cell interactions that occur in the liver to drive MASH/NASH pathology. Also, many of the animal models do not faithfully recapitulate human MASH because they lack essential metabolic perturbances or risk factors. Furthermore, as stated earlier, most of the models used have tended to be insufficient in reflecting the full pathological spectrum of MASH, especially fibrosis and cirrhosis. This latter point is a crucial deficit because of the clinical significance of fibrosis as a risk factor for MASH/NASH end stage liver disease and death. Thus, while the compelling anti-steatotic, anti-inflammatory, and pro-cell survival actions of MSC EVs are apparent, there remain only a few investigations in which its anti-fibrotic actions were assessed [34,73,144,147,153], and in none of these instances have mechanistic aspects been explored in depth.

It is important to understand whether the observed anti-fibrotic actions of MSC EVs are indirect and reflect their principal action on cells, such as hepatocytes (or possibly inflammatory cells), thus impacting upstream pathways that conspire to drive downstream fibrosis, or whether there are anti-fibrotic mechanisms that arise from the direct interactions of MSC EVs with activated pro-fibrogenic HSC in diseased livers. Indeed, HSC activation, fibrogenesis and the expression of fibrosis-associated genes are well-documented to be suppressed by MSC-EVs in vitro and in non-NASH models of liver injury. For example, the suppression of Smo Hh receptor expression and signaling in HSC by miR-486-5p EVs from human tonsil-derived mesenchymal stromal cells was attributed to the ability of the EVs to reduce fibrogenic gene expression in human LX2 HSC in vitro and to reduce fibrosis in carbon tetrachloride (CCl_4_)-treated mice [156]. EVs from miR-122-overproducing AD MSC suppressed HSC proliferation and collagen production in activated human or mouse HSC and attenuated liver damage and hepatic fibrosis in CCl_4_-treated mice [157]. In CCl_4_-induced fibrosis in mice, hUC MSC EVs reduced the expression of the collagen-crosslinking enzyme lysyl oxidase-like 2 (LOXL2) and reduced fibrosis progression, an outcome that was attributed to the ability of EV miR-27b-3p to downregulate YAP in HSC [158]. Studies of human LX2 HSC and CCl_4_-mediated liver fibrosis in mice showed that hUC MSC EVs are anti-fibrotic and induce ferroptosis in HSC while suppressing their activation [159]. This occurred through the delivery of EV-derived BECN1 to HSC, which resulted in the downregulation of the xCT/GPX4 pathway and the promotion of ferroptosis [159]. More recently, miR-378c in placental mesenchymal stem cell EVs alleviated TGF-β-mediated fibrosis in liver organoids by targeting E3 ubiquitin ligase S-phase kinase-associated protein 2 (SKP2) in HSC and causing their inactivation [160]. Collectively, these findings suggest that activated HSC are likely a legitimate target of MSC EVs in the context of NASH, although the repertoire of cell targeting by such EVs may be altered in lipid-rich livers.

Finally, while several EV components (e.g., miRs 24-3p, 96-5p, 223-3p, 627-5p, CAMKK1) have been identified as mediating some of the anti-steatotic or anti-inflammatory actions in lipid-laden hepatocytes, it unlikely that these findings are comprehensive and a much-improved understanding is needed regarding the identity of additional therapeutic components and their respective targets, especially using more relevant preclinical model systems, such as human liver organoids.

The mass manufacturing of MSC EVs is a critical challenge for translating this technology into clinical applications. A straightforward approach to increasing EV production is to scale up or scale out cell culture systems, a strategy already used in industries like monoclonal antibody manufacturing [161]. This involves employing platforms such as hyperflasks, roller bottles, and bioreactors. The primary goal is not only to maximize cell yield but also to reduce costs by minimizing manipulation time, culture duration, and the consumption of reagents. This can be achieved by increasing the surface area available for cell growth, using technologies like hollow fibers or microcarriers, which can also reduce the amount of culture medium required per cell. Another strategy is to enhance the number of EVs secreted per cell. While most cells release EVs constitutively, secretion can be further stimulated through conditions such as serum starvation, hypoxia, or various physical and chemical stresses [162,163,164,165]. Additionally, large quantities of EV-like particles can be generated by disrupting the cell membrane, although these vesicles may differ in phenotype from those naturally secreted [166]. Finally, although the data are insufficient to draw definitive conclusions, consideration must be given to the cellular source of EVs, which yields optimal therapeutic advantages. For example, as described above, ESC MSCs and their EVs appear to have superior therapeutic outcomes in non-MASLD models when compared side-by side with other EV types [151]. It has also been noted that the ease of ESC MSC culture and their high proliferation rates supports their use in future clinical applications [148]. On the other hand, relatively few studies have been performed in MASLD therapy with ESC MSC EVs as compared to hUC MSC EVs or AD MSC EVs, for which much more comprehensive data and broader sets of outcomes have been demonstrated and reproduced by several research groups. Clearly, an important future step will be the side-by-side comparison of the various type of MSC EVs in MASLD models so that the optimal therapeutic EV source can be determined.

## 6. Conclusions

MSC-EVs are attracting increased attention for their profound regenerative and anti-inflammatory properties in a wide variety of diseases across many organ systems. While MSC EVs have therapeutic properties similar to their producer cells, there are numerous advantages to the EV approach, including their ease of and stability in storage under non-cryogenic conditions, apparent low immunogenicity and toxicity, feasibility for mass-scale production, and ease of in vivo administration. In the context of MASLD, growing evidence from in vitro studies and animal models support the concept that MSC-EVs are therapeutic for steatosis, hepatocyte dysfunction, inflammation, oxidative stress, and cell survival. Some of the anti-steatotic or anti-inflammatory effects of MSC-EVs in MASH have been attributed to specific EV cargo molecules, such as miRs 24-3p, 96-5p, 223-3p, 627-5p and CAMKK1, raising the possibility that these components or their targets in the liver are new leads for therapeutic intervention, but future studies will likely identify a much broader slate of candidates. While the anti-fibrotic action of MSC-EVs in the context of MASH have also been recognized in some studies and are likely highly relevant considering the importance of clinical outcomes in patients with MASH fibrosis, a more improved understanding of the underlying therapeutic mechanisms is required. Even so, the field is now moving into an era in which MSC-EVs themselves or some of their individual or combined molecular payload components are poised to be moved into clinical trials in the hope of developing approved drugs for treating MASLD/MASH.

## Figures and Tables

**Table 1:** MSC-EV therapy in rodent models of MASH/NASH.

Type of MSCs	Species and Strain of Recipient	Liver Injury Model	Effect After MSC-EVs Treatment	EV Dose, Timing Duration, Route	Ref
Human umbilical cord-MSC	Mouse/C57BL/6 J	Methionine-choline-deficient (MCD) diet, for 6 weeks	Decreased ALT and AST; reduced TNF-a, IL-6, and IL-1b in plasma Decreased level of NF-kB protein and p-NF-kB; increased F4/80 positive cells; increased expression of CD206, arginase-1 and IL-10 in liver tissue	20 mg/kg i.v. 1st week of MCD diet. EV injection once a week	[129]
Human umbilical cord-MSC	Mouse/C57BL/6 J	20-week high fat high cholesterol (HFHC) MCD diet for 4 weeks	Serum AST and ALT levels, reduced steatosis, reversal of lipid-related gene expression (SREBP-1c, PPARα, Fabp5, CPT1α, ACOX, FAS), reduced frequency of F4/80 macrophages de-polarized macrophages in the liver	HFHC diet: 100 µg i.v. every 3 days for the last 6 weeks MCD diet: 100 µg i.v. twice a week for the last 2 weeks	[130]
Human umbilical cord-MSC	Mouse/C57BL/6 J	16-week HFD	Corrected body weights, liver weights, blood glucose and insulin, and serum AST or ALT, reduced HFD-mediated steatosis, hepatocyte ballooning, macrophage inflammation, reduced production or expression of ROS, TNFα, IL-6, ACCα, FASN, SCD1, PPARγ, NOX2, NOX4, TNF-α, IL-6, IL-1β and CCL2, and restored levels of antioxidant SOD and CAT	120 µg i.v. weekly	[131]
Human umbilical cord-MSs	Mouse/C57BL/6 J	14-week HFD	Reduced levels of steatosis, AST, ALT, liver triglycerides, total cholesterol, hepatic expression of TNF-α, IL-1, and IL-6, and restored insulin sensitivity	10 µg/g i.v. per week over the last 4 weeks	[132]
Human umbilical cord-MSC	Sprague Dawley (SD) rat	High fat high fructose for 8 weeks	Reduction in diet-induced body and liver weights, steatosis, insulin tolerance, liver injury	100 µg i.v. per rat	[133]
Rats Bone marrow MSCs	Sprague Dawley (SD) rat	HFD for 12 weeks	Suppressed steatosis, reduced hepatocyte ballooning, AST, ALT, and expression of SREBP-1, SREBP-2, ACC, CD36, decreased NAS, increased expression of PPARα and CPT1. Suppression of caspase 2, apoptosis, mitophagy	15, 30, 120 μg/kg i.v. Twice a week starting on week 7 for 6 weeks	[140]
Human Adipose-derived MSCs	Mouse/C57BL/6 J	HFD for 8 weeks	Reduced HFD-induced ALT, AST, hepatic lipid, hydroxyproline, fibrosis	100 μg i.v. twice a week starting the 2nd week of HFD diet	[144]
Human adipose tissue-MSCs	Mouse/C57BL/6 J (Mc4r-KO)	Western diet for 20 weeks with 0.3 mg/kg LPS twice a week for 4 weeks	Decreased serum ALT, crown-like structures, and fibrosis. Increased frequency of anti-inflammatory macrophages, increased expression of Mmp12, Mmp13	1, 2.5, 5 μg i.p. once 4 weeks prior	[147]
Embryonic stem cell-derived MSCs	Mouse/C57BL/6 J	200 μg Streptozotocin (STZ) 2 days after birth, HFD from 4 weeks old for 5 weeks	Reduced steatosis, hepatocyte ballooning, inflammation, fibrosis. Increased hepatic frequency of CD163+ M2 macrophages, reduced plasma IL-6	1 μg, 10 μg i.p. every other day	[34]

## Data Availability

No new data were created or analyzed in this study. Data sharing is not applicable to this article.
